# Photolysis of sulfamethazine using UV irradiation in an aqueous medium

**DOI:** 10.1039/c7ra09564c

**Published:** 2018-01-04

**Authors:** Zhigang Yi, Juan Wang, Qiong Tang, Tao Jiang

**Affiliations:** College of Chemistry, Leshan Normal University Leshan Sichuan 614004 China yizhigang117@hotmail.com; Environmental Monitoring Station of Environmental Protection Bureau of Rizhao Lanshan Lanshan Shandong 276800 China

## Abstract

Although many studies have been focused on the photochemistry of antibiotics, the roles of reactive species in photolysis and the effects of dissolved substances on antibiotic photochemical behavior have been poorly examined. The photolytic behaviors of sulfamethazine (SMN) in pure water were investigated *via* adding different scavengers to quench the active species. Results showed that decomposition of the triplet-excited state of SMN (^3^SMN*) by direct photolysis was the main path of SMN photolysis in water. Moreover, self-sensitized SMN cannot be ignored during SMN photodegradation. The main photoproducts of SMN were identified by LC-MS/MS, which indicated that SMN could not be mineralized although the photolysis under UV was effective. The effects of Cl^−^, NO_3_^−^, and fulvic acid (FA) (common substances in natural water) on SMN photolytic behaviors were also studied. The triplet-induced halogenation of SMN increases the ionic strength and reduces the ground state SMN; these are the primary causes of promotion of SMN photolysis by Cl^−^. More ˙OH produced in the presence of NO_3_^−^ could promote SMN photolysis. Competitive absorption of photons of FA with SMN and ROS scavenged by FA were the main reasons for the inhibition of SMN photolysis. The research findings are helpful for further studies on the environmental risks of ACs in natural waters and promoting the development of AC pollution treatment technology.

## Abstract 2

The role of reactive species in SMN photolysis and the effects of dissolved substances on SMN photochemical behavior.

## Graphical abstract

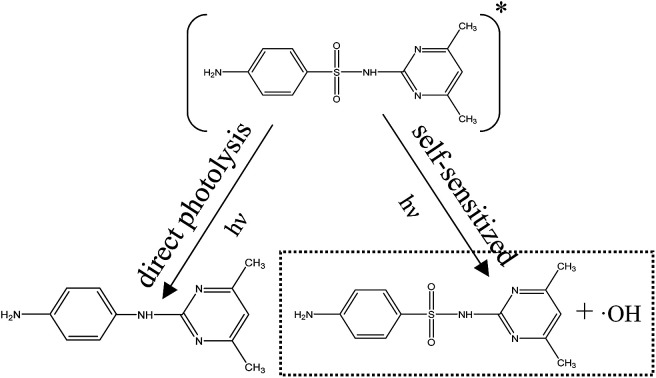

## 1. Introduction

In the last few decades, large amounts of veterinary antibiotics (ACs) have been used in animal husbandry as therapeutic medicine and feed additives for growth promotion. Moreover, one of the most popular groups of ACs, which are mainly used in livestock, are sulfonamides (SNs).^1,2^ In China, AC use in stock raising was nearly 92 700 tons, and usage of SNs was nearly 7920 tons in 2013.^3^ In the USA, 16 000 tons of ACs, including 2.3% SNs, were consumed per year. In Europe, the output of SNs ranged from 11% to 23% of the total production of ACs.^4^ SNs may be excreted from the body in their parent form without absorption and metabolism by animals. Because of their high stability and solubility, conventional sewage disposal technology used in livestock farming is not efficient for SN removal. The average removal ratio of SNs by activated sludge was found to be 24%,^1^ which meant that a mass of these drugs was introduced into the biosphere every year.^1,5^ In this case, ACs were detected in rivers, lakes, estuarine, and coastal waters many years ago.^6–8^ The highest concentration of sulfamethazine (SMN) in manure of swine farms in South China was up to 0.250 mg kg^−1^.^9^ The concentration of ACs in the aquatic environment was relatively low (ng L^−1^ to μg L^−1^),^10,11^ whereas its presence in AC manufacturing effluents might reach mg L^−1^ levels.^12^ The bacterial resistance and photo-modified toxicities of ACs could cause severe negative impact on human health and the environment.^1,13–18^ ACs have been classified as particularly dangerous pollutants for the environment.^1^

The obstinate nature of ACs residues interferes with the removal of these compounds from the environment by traditional biological treatments. Finding effective methods to eliminate the ACs residues is required for environmentally sustainable development. Heterogeneous photocatalysis, one of the typical advanced oxidation techniques (AOPs), is an efficient treatment to promote degradation of organic pollutants.^19^ Further, the performance of photocatalytic technology could be effectively improved *via* photocatalyst modification,^20^ electrochemical, microwave or ultrasonic-assisted photocatalytic technology.^21–23^ The efficiency of photocatalysis is based on the formation of reactive species through the irradiation of light energy on catalysts. During photocatalytic degradation of organic contaminants, photodegradation may play an important role, and photochemical decomposition has also been proven to be a major transformation pathway for organic pollutants in surface waters.^24,25^ Therefore, it is significant to carry out a study on photodecomposition of pollutants.

Photolysis of ACs is mainly caused by ultraviolet wavelength irradiation, and ACs can undergo not only direct or indirect photolysis, but also self-sensitized photo-oxidation.^2^ During direct photolysis, photon absorption promotes electrons from the initial ground state of ACs to produce electronically excited species in the singlet state (AS*) or triplet state (^3^AS*). On the one hand, the excited species can be decomposed into photoproducts. On the other hand, by self-sensitization, ^3^AS* can transfer the energy to the ground state of ^3^O_2_ or H_2_O to form reactive oxygen species (ROS, *e.g.*, ˙OH and ^1^O_2_).^26,27^ In addition to the direct and self-sensitized photolysis, indirect photolysis is a significant elimination pathway for many aquatic pollutants; it is initiated by ROS, formed *via* optical absorption by photosensitizers (*e.g.*, dissolved organic matter (DOM), Fe species, halide ions, and H_2_O_2_).^28–30^ However, controversy still exists about which path should play the leading role in different research. Studies indicated that many byproducts were still retained during the photolysis of antibiotics, the intermediates showed an increasing toxicity, and the solutions after photolysis still had certain residual antibacterial activity.^31–35^ Because the photolytic byproducts will be different due to different photolytic reaction paths of ACs, studies on the main factors for photolysis and the reaction paths of ACs under UV irradiation are helpful for a further study on the environmental risks of ACs in natural waters and promoting the development of AC pollution treatment technology.

In this study, we employed SMN as a proxy AC to investigate photolytic behavior under UV irradiation and detect the role of triplet-excited state and ROS in the photodegradation of ACs. Furthermore, the photodecomposition pathway of SMN was proposed. The influence of common dissolved substances in natural water on the photodecomposition of SMN was also discussed in this study.

## 2. Materials and methods

### 2.1 Chemicals

High purity standard SMN (99%), used as a target pollutant in photochemical experiments and as a standard in high-performance liquid chromatography (HPLC) analysis, was purchased from Aladdin Industrial Corporation (Shanghai, China). Acetonitrile and isopropanol were of HPLC grade and obtained from Chengdu Best Reagent (Chengdu, China). Sorbic acid (*t*,*t*-HDA, 99%), sodium chloride, sodium nitrate, fulvic acid (FA), and other chemical reagents were of analytical grade and obtained from Chengdu Best Reagent. Ultrapure water was used to prepare SMN solutions and HPLC eluent.

### 2.2 Experimental set-up

**Fig. 1:**
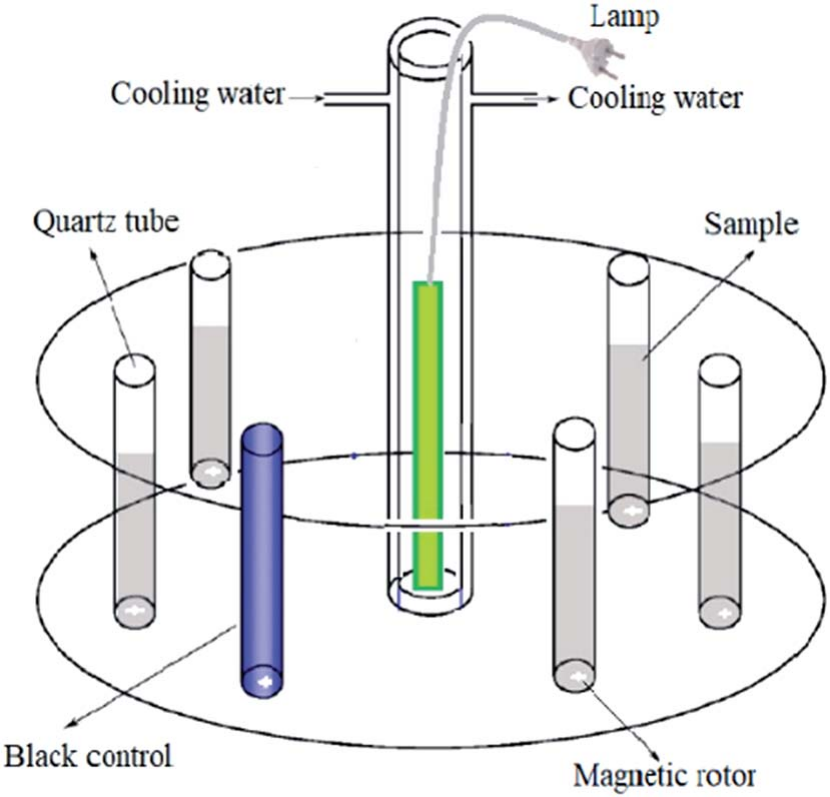
A schematic of the experimental setup.

A merry-go-round photochemical reactor with a magnetic stirrer (illustrated in Fig. 1) was employed. The high pressure Hg lamp was in a quartz sleeve and located at the center of the reactor. The temperature of the reaction system was 25 °C. The 50 mL SMN solutions were put in quartz tubes around and equidistant from the Hg lamp, irradiated under UV over a period of 60 min, and aliquots were withdrawn for analysis at scheduled time intervals. To investigate the hydrolytic degree of SMN during photolysis, non-irradiated experiments were conducted simultaneously. The SMN solutions were wrapped in an aluminum foil, and other conditions were kept the same as those for the unwrapped samples. All experiments were performed in triplicate.

### 2.3 Analytical methods

The concentration of SMN at different irradiation times was determined by HPLC (LC-2010HT, Shimadzu) coupled with UV/VIS detection (LC-UV/VIS). The parameters of the analysis were set according to the research of Batista *et al.*^2^ a C_18_ 5 μm to 100 Å column (250 mm × 4.60 mm). The detection wavelength of SMN was found to be at 268 nm. The sample injection volume was 50.0 μL. The eluents were (A) H_2_O + 0.200% acetic acid and (B) acetonitrile at 80 : 20 ratio, and the flow rate was set at 1.00 mL min^−1^. The detection and quantification limits were 0.170 mg L^−1^ and 0.500 mg L^−1^, respectively.

The products of photolysis were identified by a Shimadzu LC-20A liquid chromatography system coupled with a Shimadzu LCMS-8030 triple quadrupole mass spectrometer (LC-MS/MS). The eluent and column used were the same as those of LC-UV/VIS, but the ratio of A and B eluents was altered during the analysis: it started with 10% of B, then it increased to 60% after 10 min, then to 90% in another 8 min, and finally the content of B dropped to 10% in another 10 min and remained at this ratio until the end of the run. The detection was performed using an electrospray ionization (ESI) source operating in the positive mode. The parameters were set as follows: capillary voltage 4000 V, drying gas temperature 300 °C, drying gas flow 12 mL min^−1^, and nebulization gas 35 psi. Fragmentor voltages were adjusted between 10 and 30 V to obtain precursor ions of degradation products.

The total organic carbon (TOC) was detected by a TOC analyzer (TOC4100, Shimadzu).

## 3. Results and discussions

### 3.1 The determination of initial concentration of SMN and the light source

The concentration of SMN in the aquatic environment (ng L^−1^ to μg L^−1^) is relatively low. A pre-concentration step is required prior to quantification using HPLC.^36,37^ Although previous research has reported that the solid phase extraction (SPE) technique can improve the sensitivity of the analytical methods by HPLC, the use of SPE for quantification of sulfonamides in aqueous samples has not been examined in detail.^36^ During photodegradation of ACs, different initial concentrations of ACs caused different amounts of reactant molecules to be photo-excited; this affected the rate of photolysis, but had no impact on the reaction mechanism.^2,6^ Furthermore, Nassar *et al.*^38^ evaluated the photodecomposition of four different ACs under irradiation of sunlight and a single wavelength UV source (254 nm). The results showed that UV irradiation was effective to degrade these drugs, and photolysis of ACs under simulated solar irradiation was caused by AC absorbing the UV light from sunlight.

Due to all the abovementioned reasons, the selected initial concentration of SMN and the light source in this study were 20 mg L^−1^ and a 300 W high pressure Hg lamp, respectively. Although these two parameters were significantly different from the antibiotic environment in natural water bodies, the conclusions of this study could provide theoretical reference for the photochemical behavior of antibiotic residues in natural aquatic environments.

### 3.2 Photolysis of SMN in pure water

**Fig. 2:**
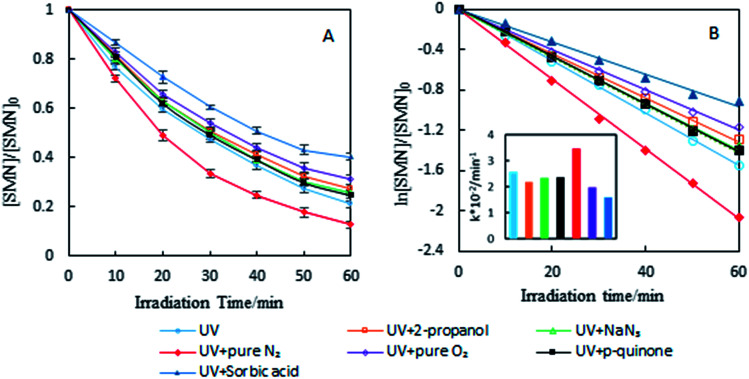
Degradation ratio (A) and kinetics (B) of SMN photolysis under different conditions.

To investigate the photolytic process of SMN, we explored SMN photolysis in pure water under 300 W UV radiation. The experiments have been carried out at pH 7 at which the neutral form of SMN dominates (>80%).^39^ Previous studies found that photo-excited pharmaceutical compounds, formed by the photosensitization process, interacted with molecular oxygen to form ˙OH, ^1^O_2_, and O_2_˙^−^.^40^ During the photolytic experiment, we selected 2-propanol, sodium azide (NaN_3_), and *p*-quinone as scavengers to quench ˙OH, ^1^O_2_, and O_2_˙^−^ respectively.^41^ SMN degradation and the logarithm of relative SMN concentration *versus* irradiation time are displayed in Fig. 2. SMN photolysis followed pseudo-first-order kinetics in pure water. The degradation ratio of SMN achieved a value of 78% after 60 min irradiation, and the degradation rate constant (*k*) of SMN was 2.58 × 10^−2^ min^−1^ in pure water. The values of *k* decreased to 2.19 × 10^−2^ min^−1^, 2.33 × 10^−2^ min^−1^, and 2.37 × 10^−2^ min^−1^ and the degradation ratios were 72%, 74%, and 75% in the presence of 2-propanol, NaN_3_, and *p*-quinone, respectively. The results indicated that ˙OH, ^1^O_2_, and O_2_˙^−^ were formed during SMN photolysis in pure water *via* the photosensitization process of SMN, and the impact of ˙OH was more noticeable than that of ^1^O_2_ and O_2_˙^−^ on the photolysis of SMN, which were consistent with the early research conclusions that hydroxyl radicals played a critical role in the photochemical transformation of organic pollutants.^42–44^ However, the ratio and rate constants (*k*) of SMN molecules had no evident decline after adding scavengers for ˙OH, ^1^O_2_, and O_2_˙^−^; this implied that direct photolysis was the main path for photochemical behavior of SMN in pure water.

To further understand the effect of self-sensitized behavior on SMN photolytic reactions, comparative trials under deoxygenation by N_2_-sparging, oxygen filling, and adding sorbic acid as a scavenger to quench ^3^SMN* were carried out in SMN photolysis. The results are also shown in Fig. 2. The value of *k* increased to 3.31 × 10^−2^ min^−1^ from 2.75 × 10^−2^ min^−1^ after deoxygenation, but decreased to 1.99 × 10^−2^ min^−1^ and 1.60 × 10^−2^ min^−1^ after oxygen filling and addition of sorbic acid, respectively (Fig. 2). Removal of ^3^O_2_ from solution by N_2_-sparging could lead to a decreased formation rate of ^1^O_2_ as well as lower quenching rate of ^3^SMN*, and oxygen filling was an opposite process to strengthen the self-sensitization. Based on these results, we could state that the self-sensitized behavior had an inhibiting effect on SMN photolysis. The reduction of *k* after adding sorbic acid further demonstrated that the steady state concentration of ^3^SMN* was very important during SMN photolysis, and the direct decomposition of ^3^SMN* was the main pathway of SMN photolysis.

To investigate the hydrolysis of SMN, non-irradiated experiments were conducted simultaneously. No obvious loss of SMN was observed in pure water solution of SMN; this implied that hydrolysis of SMN was negligible during the SMN aquatic environmental behavior.

### 3.3 Pathways of SMN photolysis in pure water

**Fig. 3:**
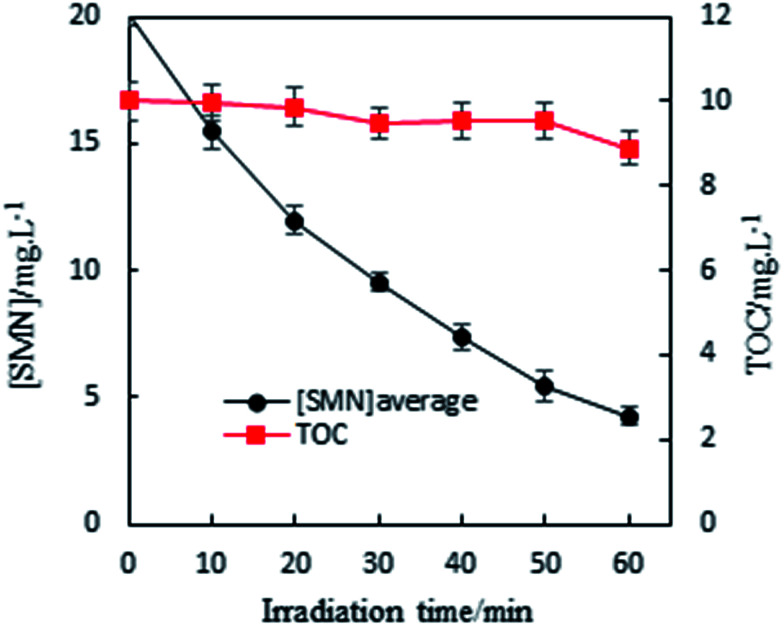
TOC with the SMN photolysis under UV irradiation.

In our experiment, we found that the main path of formation of SMN photoproducts in water was ^3^SMN* decomposition by direct photolysis. Moreover, species of ROS formed by self-sensitized SMN could degrade SMN, which could not be ignored during SMN photochemical behaviors. To determine the mineralization degree of SMN and identify the products of SMN photolysis, TOC was monitored along with SMN photolysis under UV irradiation. The solutions obtained after 60 min of SMN photodecomposition were analyzed using LC-MS/MS. As shown in Fig. 3, no significant removal of TOC was observed even after 60 min; this meant that most of the organic carbon was still retained although the SMN photolysis under UV was effective.

**Fig. 4:**
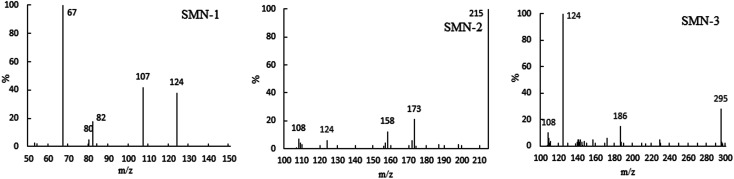
Major photolytic intermediate ion mass spectra of SMN.

**Scheme 1:**
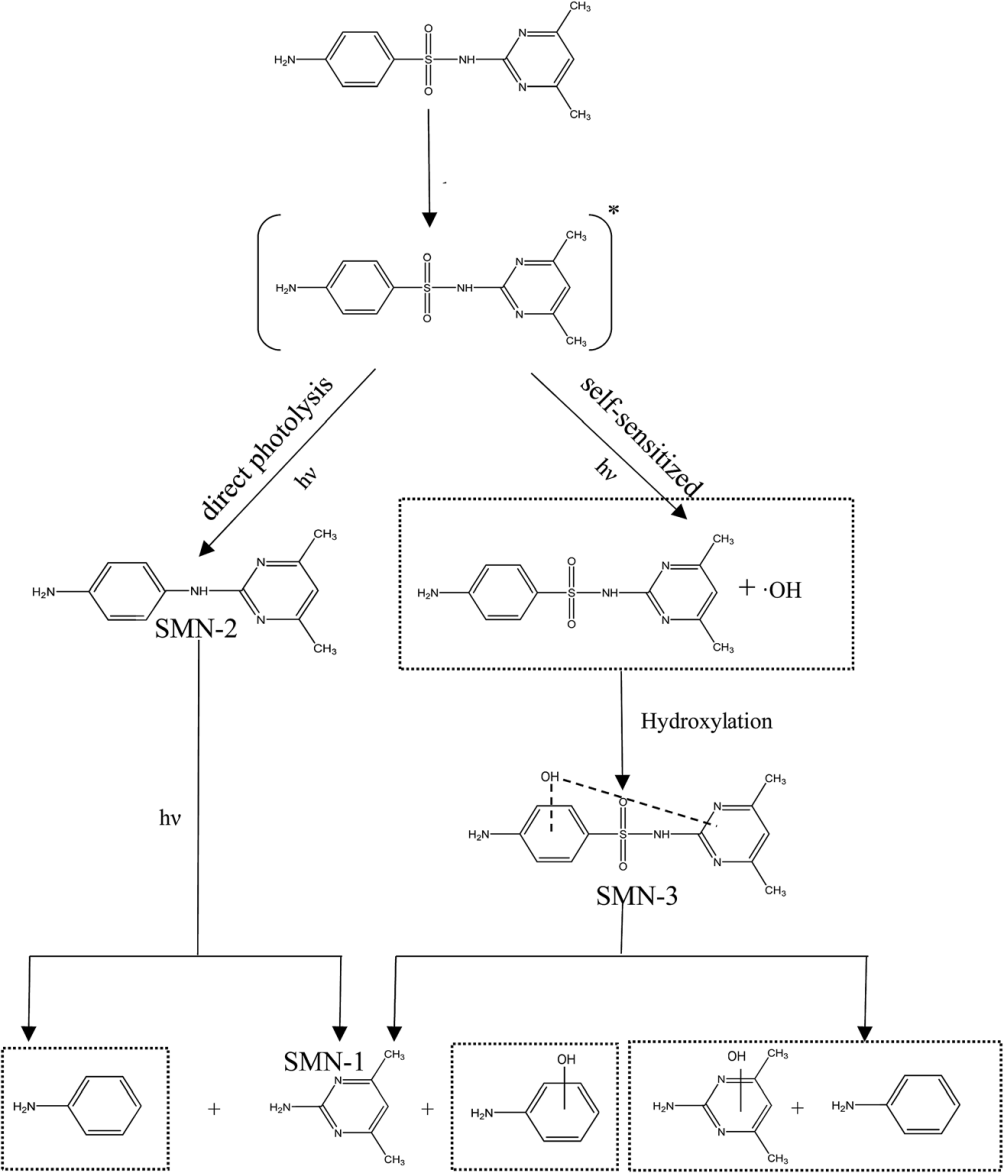
Proposed degradation pathways of SMN in pure water.

The specific ion mass spectra of major intermediates are depicted in Fig. 4. There were three main photoproducts that could be observed: SMN-1, SMN-2, and SMN-3 with *m*/*z* of 124, 215, and 295, respectively. ˙OH radicals formed during the self-sensitization process of SMN attacked the benzene ring or the dimethyl pyrimidine group of SMN; this resulted in the formation of SMN-3. SMN-2 was derived from SO_2_ removal from SMN. SMN-1 was produced from the broken carbon–nitrogen bond *via* intermediate photolysis. These results were consistent with the previous research on photolytic mechanism of sulfonamide antibiotics.^2,38^ The proposed pathways of SMN photolysis in pure water are displayed in Scheme 1.

### 3.4 Effect of soluble substances on SMN photolysis

Halide ions, nitrate, and dissolved organic matter (DOM) are ordinary soluble substances in natural waters, which can be converted into free radicals under sunlight.^6,45^ Indirect photolytic behaviors of SMN caused by soluble substances are common photodecomposition behavior of SMN. We selected Cl^−^, NO_3_^−^, and FA as probes to explore the effect of photoactive substances on SMN photolysis in water. In view of SMN having been frequently detected in both freshwater and seawater, the concentrations of Cl^−^, NO_3_^−^, and FA were set according to their content in natural waters (Table 1).^6^

**Table 1:** Concentrations of Cl^−^, NO_3_^−^, and TOC for freshwater and seawater

Parameters	Freshwater	Seawater
Cl^−^ (mM)	3.4	469
NO_3_^−^ (μM)	93.6	29.0
TOC (mg C L^−1^)	4.0	6.1

**Fig. 5:**
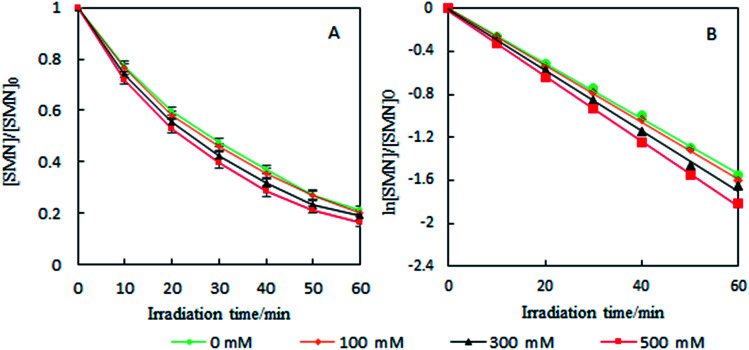
Effects of Cl^−^ on degradation ratio (A) and kinetics (B) of SMN photolysis.

**Scheme 2:**
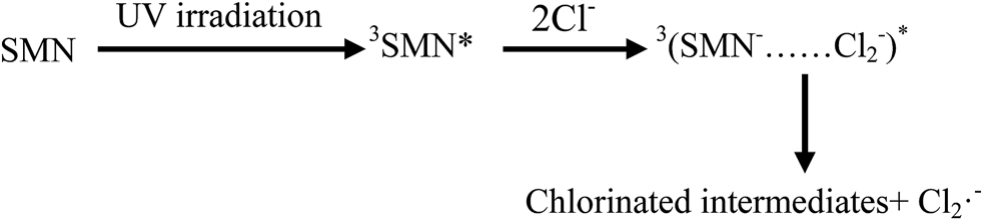
Reaction pathway of SMN induced by chloride ion.

As the content of Cl^−^ is very low in freshwater and exhibits gradient variation in estuarine waters, we set different Cl^−^ concentrations (100, 300, and 500 mM) relevant to estuarine and sea conditions^6^ to explore the effects on the photolysis of SMN. Experiments showed that the *k* values of SMN photolysis increased with an increase in Cl^−^ concentration (Fig. 5). In pure water, the main photolysis pathway is ^3^SMN* disintegration to photoproducts by direct photolysis. Hence, the steady state concentration of triplet-excited state (T_ss_) is very important for degradation. Previous studies indicated the T_ss_ of ^3^SAs* was higher at a higher ionic strength.^46,47^*E*_T_ was employed to evaluate the electron accepting ability of the ^3^SAs*.^48,49^ SMN exists in natural water (pH = 6.5–8.5) as two different species: neutral state (SMN^0^) and anionic state (SMN^−^).^39^ The *E*_T_ values are 2.45 V and 2.25 V, which let ^3^SMN* oxidize Cl^−^ [*E*(Cl_2_˙^−^/Cl^−^) = 2.0 V].^6^ Thus, the deactivation of ^3^SMN* induced by Cl^−^ may lead to the formation of Cl_2_˙^−^ and halogenated intermediates *via* the pathway demonstrated in Scheme 2. Triplet-induced halogenation of SMN will increase the ionic strength effect, and at the same time reduce the ground state SMN; this can explain why the rate of SMN photolysis is faster in the presence of Cl^−^ as compared to that in the presence of pure water.

The role of NO_3_^−^ was paradoxical: either its absorption (200–400 nm) covering the SMN absorption range (200–330 nm) resulted in a decrease of photolytic rate of SMN or produced reactive species (*e.g.*, ˙OH, NO_2_˙) *via* sensitization effects (eqn (1)–(3)); this could increase the photolytic rate of SMN.1
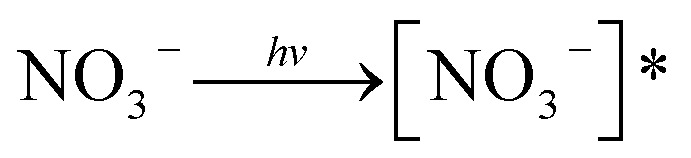
2
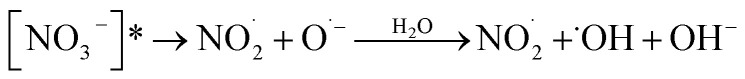

3 [NO_3_^−^]* → NO_2_^−^ + O(^3^P)

**Fig. 6:**
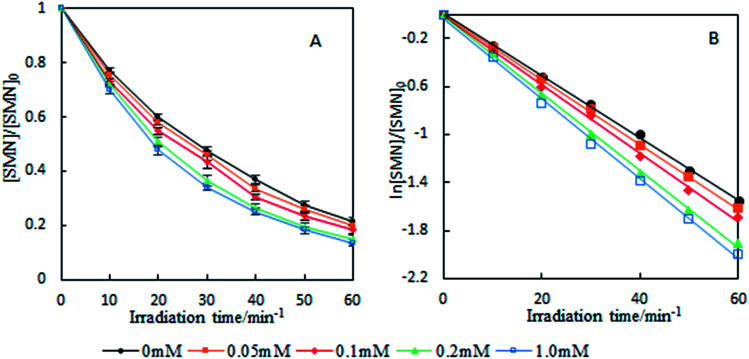
Effects of NO_3_^−^ on degradation ratio (A) and kinetics (B) of SMN photolysis.

**Fig. 7:**
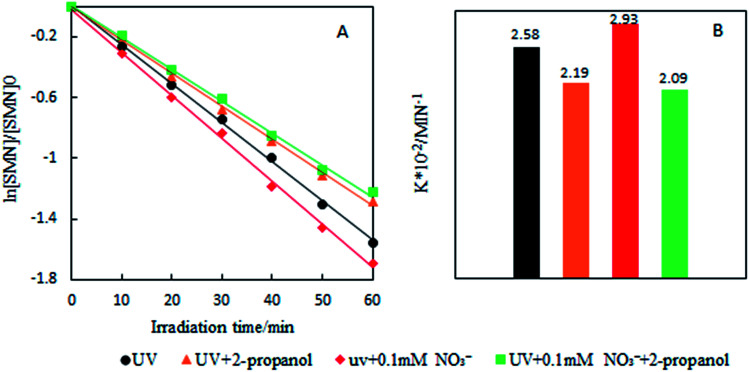
The *k* values of SMN photolysis after adding NO_3_^−^ and 2-propanol.

Our experimental results are shown in Fig. 6. In the concentration range of NO_3_^−^ in natural water, the existence of NO_3_^−^ led to the increase of SMN photolytic rate. To know whether ˙OH formed by NO_3_^−^ sensitization played an important role during SMN photolysis, we added 2-propanol to the reaction system; the experimental findings are exhibited in Fig. 7. The *k* value of SMN photolysis decreased than that in pure water. The experiment confirmed that more production of ˙OH in the presence of NO_3_^−^ as compared to that in pure water was the main cause of NO_3_^−^ promoting the SMN photolysis.

**Fig. 8:**
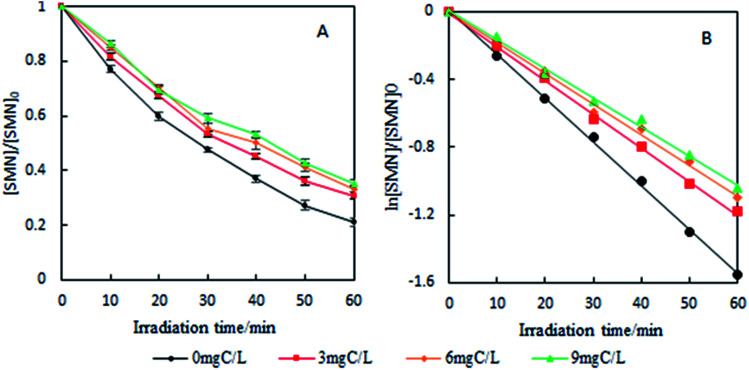
Effects of FA on degradation ratio (A) and kinetics (B) of SMN photolysis.

FA is a ubiquitous DOM in natural waters that has strong photochemical activity. In this study, following the concentration of DOM in natural waters,^50^ different concentrations of FA (3.0, 6.0, and 9.0 mg C L^−1^) were added to pure water to investigate the effect of DOM on the photolysis of SMN. As shown in Fig. 8, photolysis rate constants of SMN decreased with an increase in FA concentration.

These conclusions were not consistent with previous studies. Others found that FA formed the excited state ^3^FA* and ROS under irradiation, which further induced the degradation of organic compounds and was a significant promoter of organic compound photolysis.^51^ The photolysis pathway of FA in SMN solution is displayed in eqn (4)–(6).4
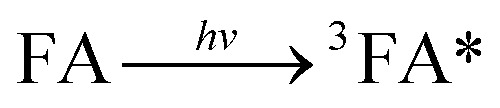
5
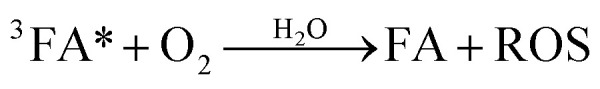

6 FA + ROS → photolytic products

In our case, the inhibiting effect of FA on SMN photolysis might have two factors: one, the steady state concentration of ^3^SMN* was very important for SMN photolysis; the direct decomposition of ^3^SMN* has been proven to be the main pathway of SMN photolysis. Therefore, the competitive absorption for photons (200–400 nm) by FA could form ^3^FA*, which inhibited the production of ^3^SMN*. Another, the ^3^FA* was quenched by dissolved oxygen in the water. This process takes ^3^FA* back to the ground state (FA). FA not only acts as a ROS scavenger,^52^ but also inhibits SMN from absorbing the photons.

## 4. Conclusion

In this study, we investigated the photolytic behaviors of SMN *via* adding different scavengers to quench active species in pure water. The research indicated that ^3^SMN* played an important role in SMN photolysis: the main photolytic path of SMN in water was ^3^SMN* direct photolysis. Moreover, ˙OH formed by self-sensitized SMN could degrade SMN, which could not be ignored during study of SMN photochemical behaviors. The main photoproducts of SMN were identified by LC-MS/MS, which showed that SMN could not be mineralized although photolysis under UV was effective. *Via* studying the effects of ordinary soluble substances in natural water on SMN photolysis, we also found that the rate of SMN photolysis was faster in the presence of Cl^−^ and NO_3_^−^. The triplet-induced halogenation of SMN increased the ionic strength and reduced the ground state SMN; these were the primary causes of promotion of SMN photolysis by Cl^−^. More ˙OH produced in the presence of NO_3_^−^ could be of advantage to SMN photolysis. A low concentration of FA in water inhibited SMN photolysis. Competitive absorption of photons by FA with SMN and ROS scavenged by FA were considered to be the main reasons. The environmental risks of intermediates during SMN photolysis in waters must cause concern.

## Conflicts of interest

There are no conflicts of interest to declare.

## Supplementary Material
